# Stiff left atrial syndrome with pulmonary veins occlusion after percutaneous radiofrequency ablation: a life-long complication that can lead to heart transplantation

**DOI:** 10.1186/s13019-023-02193-6

**Published:** 2023-05-16

**Authors:** Rani Kronenberger, Kaoru Tanaka, Carlo de Asmundis, Mark La Meir

**Affiliations:** 1 Cardiac Surgery Department, Universitair Ziekenhuis Brussel, Laarbeeklaan 101, Brussels, 1090 Belgium; 2 Radiology Department, Universitair Ziekenhuis Brussel, Laarbeeklaan 101, Brussels, 1090 Belgium; 3 Heart Rhythm Management Centre, Universitair Ziekenhuis Brussel, Laarbeeklaan 101, Brussels, 1090 Belgium

**Keywords:** Atrial fibrillation, Ablation, Pulmonary vein occlusion, Stiff Left Atrial Syndrome

## Abstract

**Background:**

Stiff left atrial syndrome (SLAS) and pulmonary vein (PV) occlusion are rare yet potentially major life-long complications after radiofrequency ablation for atrial fibrillation. While mostly controlled by medical management, SLAS can progress to refractory congestive heart failure. Treatment of PV stenosis and occlusion remains a challenging problem with ongoing risk for recurrence regardless of techniques employed. Herein we present the case of a now 51-year-old male with acquired PV occlusion and SLAS who, over the course of eleven years, despite multiple interventions, ultimately required heart transplantation.

**Case presentation:**

After undergoing three radiofrequency catheter procedures for paroxysmal atrial fibrillation (AF), a hybrid ablation was planned due to reappearance of symptomatic AF. Preoperative echocardiography and chest computed tomography (CT) revealed an occlusion of both left PVs. Furthermore, left atrial dysfunction, high pulmonary artery and pulmonary wedge pressures were diagnosed as well as an important reduction of the left atrial volume. The diagnosis of stiff left atrial syndrome was made. Primary surgical repair of the left-sided PVs was performed using a pericardial patch as a tubular neo-vein, combined with cryoablation in the left and right atrium to treat the patient’s arrhythmia. Initial results were favorable, however, after two years the patient experienced progressive restenosis with hemoptysis. Therefore, stenting of the common left PV was performed. Over the years, progressive right heart failure with severe tricuspid regurgitation developed, despite maximal medical therapy, which led to the need for heart transplantation.

**Conclusion:**

The impact of PV occlusion and SLAS after percutaneous radiofrequency ablation can be lifelong and devastating for the clinical course of the patient. Since the presence of a small left atrium could be an important predictor for SLAS in case of redo ablation, preprocedural imaging should guide the operator to an algorithm of a decision-making containing lesion set, energy source, and safety of re-ablation.

## Background

Catheter ablation has emerged as the cornerstone of therapy for drug-refractory AF and as a first-line option for paroxysmal AF. Pulmonary vein stenosis (PVS) is a major complication of catheter ablation (0.3-3.4%) [1]. It is defined as a reduction of the PV diameter and is classified as mild (< 50%), moderate (50–70%) and severe (> 70%). Its incidence has decreased by avoiding ostial isolation. Nevertheless, when severe PVS occurs, it remains an important problem with clinical symptoms like chest pain, dyspnea, cough, fatigue, decreased exercise tolerance, recurrent lung infections, symptoms of pulmonary hypertension and hemoptysis. Balloon angioplasty (BA), often with stenting, is the most common treatment.

Stiff left atrial syndrome is a rare complication due to left atrial scarring after radiofrequency ablation (RFA). This leads to left atrium (LA) volume reduction and reduced compliance. Although the diagnosis is often delayed, presence of dyspnea, congestive heart failure, pulmonary hypertension (PH), large V waves on pulmonary capillary wedge pressure or LA pressure tracings should increase awareness. Most patients are successfully treated with diuretics.

Herein we present a case of left PV occlusion combined with SLAS ultimately leading to heart transplantation.

## Case Presentation

**Fig. 1:**
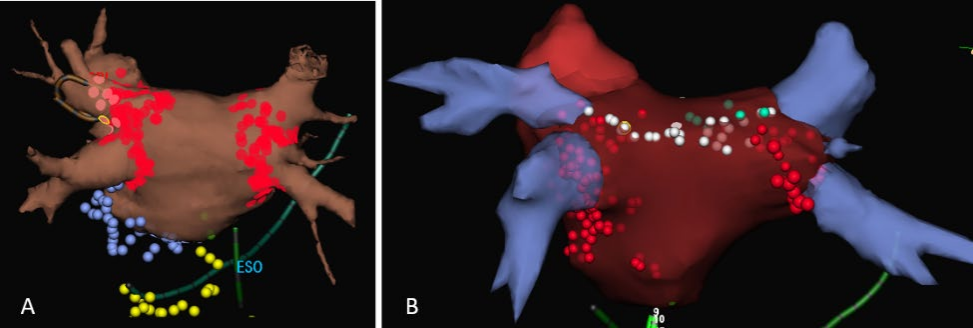
Electroanatomical mapping with 3D reconstruction of the left atrium and ablation lines. (A) First procedure. Left and right PV isolation, mitral isthmus line and defragmentation of the coronary sinus. (B) Second left atrial ablation with creation of a roofline

A 40-year-old Caucasian male was referred to our institution for a hybrid AF procedure after three percutaneous radiofrequency (RF) ablations. He underwent a first AF ablation in June 2010 for drug-refractory paroxysmal AF consisting of RF pulmonary vein isolation, mitral isthmus ablation and defragmentation of the coronary sinus. An additional right cavo-tricuspid isthmus ablation was performed in October 2010, followed by a roofline in March 2011 (Fig. 1).

The procedures and the postoperative course were uneventful apart from recurrence of symptomatic arrhythmia. He had no history of structural heart disease, normal left and right ventricular ejection fraction, normal coronary arteries, no diabetes and no sleep apnea syndrome.

**Fig. 2:**
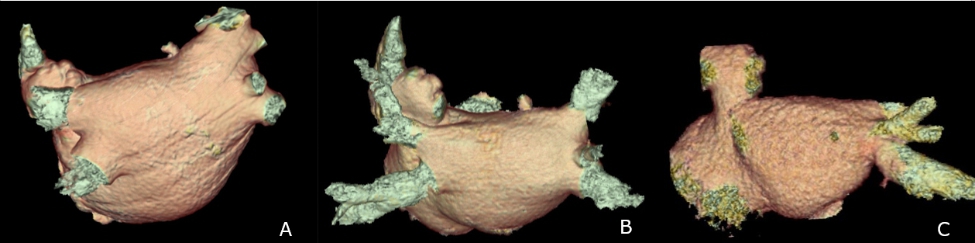
Cardiac CT with 3D reconstruction showing progressive reduction of left atrial volume. (A) LA volume 141 ml before ablation. (B) LA volume 90 ml after first ablation procedure. (C) LA volume 51 ml after third ablation procedure with occlusion of the left PVs

During admission, a preoperative transthoracic echocardiography revealed a small left atrial diameter of 29 mm. A contrast-enhanced chest computed tomography (CT) revealed occlusion of the left PVs and a major reduction of the LA volume from 141 ml pre-ablation (CT: June 2010) to 51 ml after the third ablation (CT: November 2011) (Fig. 2A-C).

**Fig. 3:**
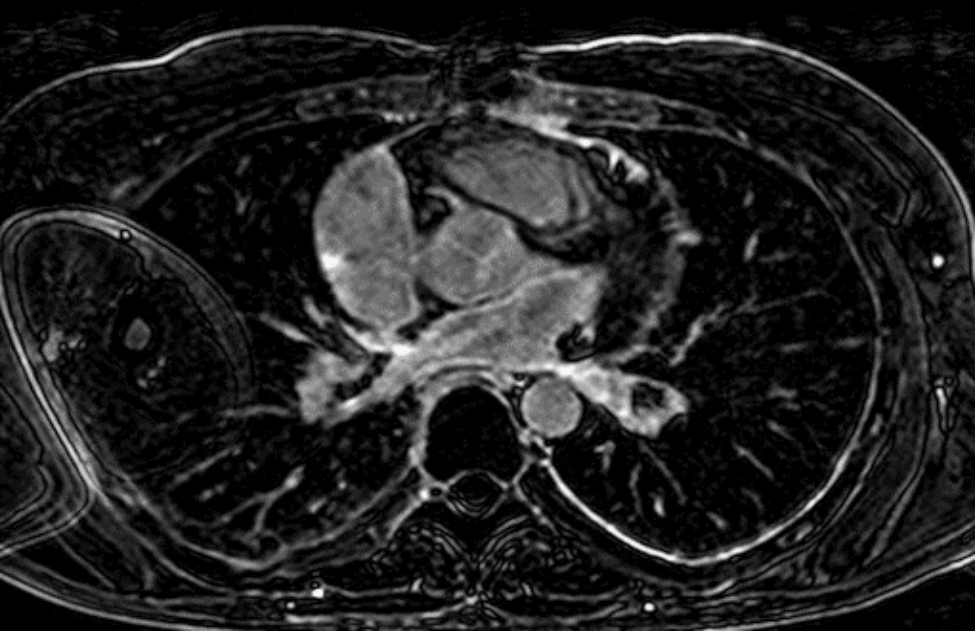
Cardiac 3D late Gadolinium magnetic resonance imaging showing an extremely small left atrium with extensive fibrosis

Cardiac magnetic resonance imaging revealed an extremely small left atrium with extensive fibrosis (Fig. 3).

A pulmonary arteriogram confirmed total occlusion with a length of approximately 1 cm and the absence of reasonably sized veins distal to the occlusion. Therefore, surgery was the treatment of choice rather than BA with stenting (November 2011).

Surgery was performed through median sternotomy. The small fibrotic left atrium was opened over the Waterston’s groove and the ostiae of the PVs were inspected. Both left PVs were occluded. The right veins were not significantly reduced. The left pleura was opened confirming continuation of the strictures of the PVs over their entire length. Both PVs were then longitudinally incised at the hilum. The remaining diameters at this level were respectively 3 and 4 mm. Surgical left PV reconstruction with a pericardial patch plasty was performed. A neo-vein was created using a 2.5 by 5 cm bovine pericardial patch starting from distal (intrapleural) to proximal, including the occluded part of the PVs as the posterior wall. This neolung vein was implanted in the left atrium as a common ostium. Concomitant left-sided cryo lesions (roof and inferior line) and a right-sided cryo-maze were made. A left atrial appendage (LAA) exclusion or resection, which is often done as an adjuvant therapy in surgical AF procedures, was not performed to avoid further reduction of the LA volume. After weaning, transesophageal echocardiography showed good patency of the (neo)pulmonary vein with increased flow rates related to the small venous diameters at the level of the hilum.

**Fig. 4:**
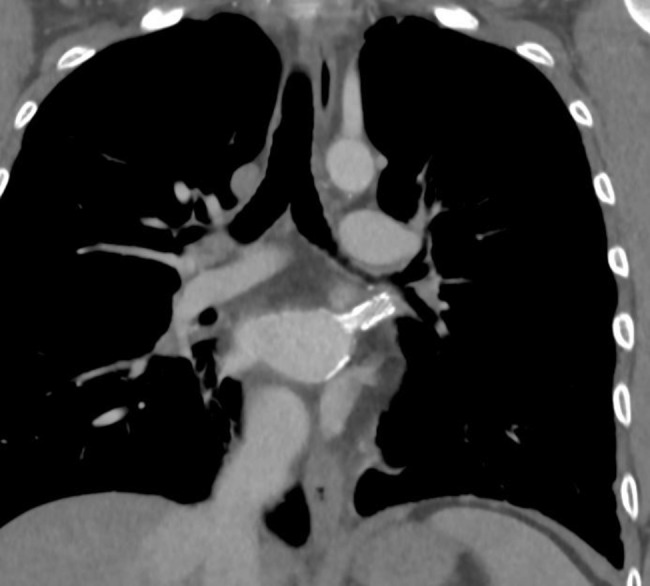
CT showing the common ostium of the left PVs after stenting

The patient was extubated within 4 h and was discharged on day 7 on coumadin and aspirin. At 3 months follow-up, the patient was free of symptoms. Transesophageal echocardiography revealed right ventricular (RV) dysfunction with patent PVs. Over the following months, symptom burden due to progressing tricuspid regurgitation (TR) increased and diuretics were started. Two years later, after an episode of hemoptysis, a CT showed stenosis of the common ostium left PVs. A successful additional BA and stenting of the left PV was performed using a Valeo vascular stent (Fig. 4).

In the following years, despite maximal right heart failure treatment, symptoms and RV failure steadily progressed. Eleven years later, he received a heart transplantation, with removal of the stent and reimplantation of the left PVs.

## Discussion and conclusions

To the best of our knowledge, this is the first report describing a patient with pulmonary veins occlusion and stiff left atrial syndrome after percutaneous RFA leading to heart transplantation.

The decision to perform a redo catheter ablation due to recurrence of arrhythmia should not only be guided by the potential outcome, but also by the risk of complications. Since it is unclear whether a subgroup of patients is more prone to develop PV stenosis or SLAS, preprocedural imaging should be performed. Reduction of PVs diameter or excessive LA volume reduction should caution the operator. Interestingly, in a published series on right heart dysfunction post RFA, with findings of increased left atrial pressure and pulmonary hypertension, the discovery of PV stenosis or occlusion automatically excludes the patient from the diagnoses of SLAS [2]. By definition, PVS precludes SLAS. We believe that both complications were present in our case. However, after treatment of the left PV occlusions, it was mainly the SLAS that became responsible for the progressive right heart failure.

Catheter ablation for treatment of AF has evolved from ostial PV isolation to more extensive LA ablation to reduce the risk of PVS and improve sinus rhythm outcome. Interestingly, this strategy could increase the risk for development of SLAS. A more antral isolation may lead to the formation of substantial left atrial scarring, decreased left atrial volume, and impaired left atrial diastolic function.

Monopolar radiofrequency isolation of the PVs and linear lesions in the left atrium can produce luminal narrowing or occlusion which is predominantly due to intimal thickening, thrombus formation, endocardial contraction, and proliferation of elastic laminae [3]. Klimek-Piotrowska et al. described the normal distal pulmonary vein anatomy in autopsied adult human hearts. They found a mean ostium-to-last-tributary (closest to the atrium) distance of 15.1 ± 4.6 mm for left superior PV, 13.5 ± 4.0 mm for left inferior PV, 11.8 ± 4.0 mm for right superior PV and 11.0 ± 3.7 mm for right inferior PV [4]. There is a tendency to accept that acquired PVS is an ostial problem. Most published reports do not describe stenosis length and focus primarily on the remaining PV diameter. Packer et al. challenged this concept in a study on 23 PVS patients having undergone PV isolation. They measured a mean stenosis length of 19 ± 6 mm (range: 7 and 35 mm). Twelve of the 23 patients (52%) had undergone a second ablation due to recurrence of AF, whereas five patients had undergone a third ablation procedure. A total of 34 vessels developed stenosis, of which 25 had been ablated once and nine ablated twice [5]. In a randomized head-to-head comparison of acute and chronic pulmonary vein stenosis for cryoballoon versus RF ablation, Watanabe et al. concluded that acute reduction in the luminal area of the left PVS was significantly smaller in the CB arm (N = 25) than the RF arm (N = 25). There was no difference in the extent of PV stenosis 3 months post ablation between the arms (0–25% stenosis, 90% vs. 88%, 25–50% stenosis, 10% vs. 12%, > 50% stenosis, both 0%; P = 0.82). A greater acute PV narrowing was likely to lead to chronic stenosis in the radiofrequency arm. Furthermore, to avoid PVS, a wider antral isolation is preferred, which as aforementioned could increase the risk for SLAS [6]. In contrast to radiofrequency ablation, cryo energy preserves the matrix of the cells, explaining these findings. This is the reason why we chose to complete the lesion set with cryo energy in this patient with preexisting SLAS.

Uniform treatment guidelines for acquired PVS cases have yet to be established. An invasive therapy is proposed in patients with more than one stenotic PV > 70% and with important clinical symptoms. Management comprises interventional transcatheter and surgical therapies. BA often combined with stenting is the preferred strategy. However, outcomes remain suboptimal due to high risk of restenosis. In a meta-analysis comparing efficacy and safety of BA alone versus stenting, by Buiatti et al., a total of 4 studies treating 315 PVS in 188 patients (BA, n = 171 versus stent, n = 144 PVS) were considered. After a median follow-up of 32 months, the overall incidence of restenosis was 46%. A percutaneous therapy with BA was associated with a higher risk for restenosis requiring repeat intervention compared to stenting [7]. In a review on treatment of congenital and acquired pulmonary vein stenosis, Suntharos et al. concluded that the arbiter of success for any intervention is the upstream size of the vein, both for percutaneous and surgical interventions, and for congenital and post-ablation acquired PVS. With intrinsically small pulmonary veins, repeat interventions appear to be the only answer to maintain patency [8]. This was also seen in our case where the remaining diameters at the level of the hilum were respectively 3 and 4 mm. In very severe stenosis and occlusion of PVs, surgical repair by grafting or reconstruction by pulmonary angioplasty, patch plasty and an in situ sutureless patch using the pericard or the LAA can be performed [9]. However, surgical treatment of PVS also remains a challenging problem with risk for recurrence of stenosis regardless of surgical technique employed. Frequently, the extent of fibrosis in the cases selected for surgery typically goes beyond the carina of the PV into the hilum of the lung. Therefore, the remaining diameter at the level of anastomoses at the last tributary lung vein is small. Given the length and severity of PVS distal to the occlusion in this case, primary surgical repair by a tubular reconstruction of both veins using pericardial patch, creating a common ostium at the atrium, was chosen. The LAA was not used as a patch to avoid further reduction of LA volume. Although restenosis did occur, stenting of the common ostium could safely be performed.

SLAS, defined as symptomatic LA diastolic dysfunction associated with PH in the absence of mitral valve disease or PVS, is an uncommon yet severe complication. It is characterized by high pulmonary artery wedge pressures and left atrial diastolic pressure. In a study by Gibson et al. this syndrome was detected in 1.4% patients post RFA. A redo procedure was not a risk factor for the development of SLAS. The authors concluded that only LA scarring, diabetes and obstructive sleep apnea were more frequently observed among those who developed PH [10]. However, most papers agree that the risk of restenosis remains highest in those with a history of multiple AF ablations and that there is often a delay of weeks to months between the ablation procedure and clinical symptoms initiating the diagnosis. Interestingly, SLAS has never been described after cryo procedures. Ablation and sinus rhythm often significantly decreases LA size and volumes due to atrial remodeling without adversely affecting LA diastolic and systolic function. In our case, excessive scar tissue formation reduced the LA volume from 141 ml pre-ablation to 51 ml post-ablation. Since the presence of a small left atrium is an important predictor for SLAS in case of redo ablation, preprocedural imaging in this case should have guided the operator and question the safety of re-ablation. Even though the PVs stenosis was treated, SLAS evolved. The initial improved quality of life progressively became worse when right heart failure installed over the course of years with a severe TR, refractory to maximal diuretic therapy. Interatrial septostomy for controlled left atrial decompression as described by other groups as a treatment of heart failure was not considered since this patient’s heart failure was primarily related to a severe RV dilation and TR [11]. A heart-lung transplantation could be avoided since the left lung recovered after hybrid treatment of the left pulmonary veins occlusion by angioplasty and stenting.

To conclude, pulmonary vein occlusion and stiff left atrial syndrome after percutaneous radiofrequency ablation have a life-long high morbidity, are potentially fatal and demand a complex treatment strategy. Although further studies are required to identify which ablation techniques and energy sources might avoid these complications, the presence of preexisting reduction of PVs and moderate PH with evidence of LA volume reduction should caution the operator when considering redo-ablation.

## Data Availability

Not applicable.

## List of Abbreviations

AF: Atrial fibrillation
BA: Balloon angioplasty
CT: Computed tomography
LA: Left atrium
LAA: Left atrial appendage
PH: Pulmonary hypertension
PVS: Pulmonary vein stenosis
PV: Pulmonary vein
RF: Radiofrequency
RFA: Radiofrequency ablation
RV: Right ventricular
SLAS: Stiff left atrial syndrome
TR: Tricuspid regurgitation
